# Cancer Genetic Predisposition and Clinical Applications—A Narrative Review on Germline Genetic Testing, High-Risk Cancer Surveillance and Management

**DOI:** 10.3390/genes17060648

**Published:** 2026-05-31

**Authors:** Xia Wang

**Affiliations:** College of Medicine, Central Michigan University, Mt. Pleasant, MI 48601, USA; wang23x@cmich.edu; Tel.: +1-(989)746-7500

**Keywords:** cancer genetics, tumor suppressor gene, germline genetic testing, variant classification, cancer predisposition, hereditary cancer syndrome, high-risk cancer screening, cancer prevention, polygenic risk score (PRS)

## Abstract

Understanding germline genetic variation is essential for improving human cancer care. Cancer predisposition genetic testing has become a part of the landscape of healthcare. Clinical guidelines have been established to identify individuals with monogenic risk, support variant classification, and guide enhanced cancer surveillance and prevention strategies. However, genetic mechanisms, cancer syndromes, genetic testing, patient education, and high-risk cancer management are often addressed in separate professional domains leading to limited cross-disciplinary understanding and confusion. A review tailored to a broad spectrum of clinicians is necessary to synthesize information, connect key concepts, and clearly define the principles and reasoning underlying recommended practice. Advanced genetic technology identified numerous genes and countless pathogenic variants contributing to a wide range of cancer predispositions. Rapid and accurate next-generation sequencing has enabled the routine use of multi-gene panel testing to stratify cancer risk. In precision cancer therapies, tumor genomic profiling frequently uncovers not only somatic alterations but also germline mutations, revealing additional cancer risk for the patients and their biological relatives. Beyond monogenic risks, the cumulative effect of numerous common polygenic factors can also significantly influence cancer susceptibility. Despite major advances in integrating germline genetic information into cancer care, substantial challenges remain in variant interpretation, precise risk stratification, and implementing personalized screening and prevention strategies. Using several cancer predisposition syndromes as examples, such as breast and ovarian cancer syndrome, Lynch syndrome, and Li-Fraumeni syndrome, the review provides a high-level overview of key concepts, the evolution of knowledge and technology, and the rationale underlying the current clinical management strategies.

## 1. Introduction

Germline genetic variation contributes significantly to tumorigenesis, tumor progression, and therapeutic response in cancer. Approximately 8% of cancers have monogenic etiologies [1]. Over the past decades, substantial advances have been made in leveraging germline information to improve cancer care. Numerous high and moderate-penetrance variants, particularly in tumor suppressor genes, have been identified as drivers of inherited cancer susceptibility. Therefore, clinical genetic testing for monogenic pathogenic variants (mutations) has been widely adopted in risk-stratified cancer screening and prevention.

However, hereditary cancer syndromes, genetic testing, testing candidate identification, patient education, and high-risk cancer management are often addressed in separate professional domains, leading to siloed education and limited cross-disciplinary understanding. Consequently, specialists in one area may lack comprehensive familiarity with related fields. Identifying high-risk individuals and initiating the evaluation process relies on a broad range of clinicians. This review aims to provide perspectives on how genetic research has shaped current clinical strategies for identifying, monitoring, and preventing hereditary cancer. Rather than offering an exhaustive discussion of individual high-risk cancer genes or related management strategies, this provides a high-level overview of essential concepts, using representative examples to illustrate core principles.

This review traces the timeline of research on genetic cancer predisposition and its translation into clinical practice. It covers the period from 1990, when *BRCA1* was first identified as a clinically relevant tumor suppressor gene, to April 2026, when updated clinical guidelines were last accessed. References are selected to highlight milestones in tumor suppressor gene discovery, genetic testing, and cancer risk management.

Clinical practice guidelines have been developed across different countries and medical specialties, addressing numerous genes and their associated cancer risks based on clinical evidence and expert consensus. The review discusses cancer–gene associations and clinical management strategies, primarily based on the United States National Comprehensive Cancer Network (NCCN) Practice Guidelines (https://www.nccn.org/, accessed 25 April 2026) [2] unless otherwise noted. Guidelines under each subject evolve and update regularly (once a year, sometimes more than once a year) to keep up with the advances of knowledge and evidence. Specifically, the article cited NCCN Genetic High-Risk Assessment guidelines for “Breast, Ovarian, Pancreatic and Prostate” (Li-Fraumeni syndrome included) was Version 3.2026, and the cited guidelines for “Colorectal, Endometrial and Gastric” was Version 1.2025. Each hereditary cancer risk affects a relatively small proportion of the population over a lifetime. The volume of data has limited the feasibility of conducting large randomized controlled trials to definitively evaluate the current management approaches. Consequently, most NCCN recommendations are based on uniform expert consensus and lower-level evidence. These include phase II trials, cohort studies, and institutional experience. The NCCN classifies such evidence as Category 2A. The cancer–gene association is supported by either “strong” or “very strong” evidence, derived from multiple studies or well-replicated data of large studies.

Considering disease prevalence, mutation frequency within populations, and the strength of available evidence, three conditions are selected to illustrate current approaches for identifying hereditary cancer risk and risk-reducing strategies in accordance with U.S. guidelines. These conditions include BRCA1/2 associated breast and ovarian cancers, mismatch repair (MMR) deficiency associated Lynch syndrome, and TP53-related Li-Fraumeni syndrome (LFS). LFS is discussed as an example of a condition with exceptionally high risk, but suboptimal management strategies.

Eleven key areas are addressed: high-risk tumor suppressor genes; criteria for germline genetic testing; testing methodologies; variant classification; advanced screening for high-risk individuals; cancer prevention strategies; hereditary tumor syndromes with both cancer and noncancerous manifestations; gene penetrance; polygenic risk assessment; integration of somatic genetic testing; gene-targeted therapies; and considerations under the Genetic Information Nondiscrimination Act (GINA).

## 2. Tumor Suppressor Genes Responsible for Inherited Risk of Cancer

Breast cancer is the most common cancer among women in the United States. Approximately one in eight women will develop the disease during their lifetime, with most cases diagnosed in the sixth or seventh decade of life. However, about six in 1000 women develop breast cancer before the age of 40, prior to the start of routine population-based screening [3]. In the 1980s, growing attention and research began to focus on families with multiple young women succumb to breast cancer. In 1994, a linkage study and molecular cloning identified *BRCA1* as the first gene responsible for autosomal dominant inherited susceptibility to breast cancer [4]. Women in these kindreds were also found to have a markedly increased risk of ovarian cancer [5]. Soon after, in 1995, the *BRCA2* gene was identified as another gene responsible for similar risks [6]. Additionally, families with *BRCA2* mutations were shown to have a higher incidence of male breast cancer, prostate cancer and pancreatic cancer [7]. Molecular research coined *BRCA1* and *BRCA2* as tumor suppressor genes participating in homologous recombination repair (HRR) of double-stranded DNA breaks. The germline dysfunction of BRCA1/2 contributes to a lifetime predisposition to cancer [8,9,10]. Subsequently more tumor suppressor genes were identified and confirmed to be associated with hereditary risk for cancers.

Lynch syndrome was initially named hereditary nonpolyposis colorectal cancer (HNPCC) after *MLH1* and *MSH2* gene mutations were linked to colorectal cancers (CRC) diagnosed before 30-years of age [11]. The DNA mismatch repair protein MutL was first discovered in bacteria decades earlier. Its human homolog (hMLH1) was later recognized as the germline cause of HNPCC in affected families. The group of MMR genes associated with autosomal dominant CRC risk has since expanded to four, *MLH1*, *MSH2*, *MSH6* and *PMS2*. As the spectrum of associated malignancies broadened beyond CRC, the condition was later recognized as Lynch syndrome, encompassing cancers of the endometrium of the uterus, ovary, urothelium of the upper urinary tract, and sebaceous gland of the skin (i.e., sebaceous carcinoma) [12].

Another less common but highly aggressive cancer syndrome is Li-Fraumeni syndrome, where very early onset malignant neoplasms can arise from a wide range of tissue organs. The causative gene is *TP53*, a critical tumor suppressor governing the timing of DNA repair, cell cycle arrest, senescence, or apoptosis. A carrier with a germline mutation in *TP53* is at high risk of malignant neoplasia in the brain, blood, bone, breast, gastrointestinal (GI) tract, endocrine, as well as a group of soft tissue cancers called sarcoma [13]. *TP53* mutations are among the most powerful drivers of tumorigenesis. When sporadically occurring in tissue, deleterious variants are called somatic mutations. *TP53* somatic mutations are present in approximately 50% of all human malignancies and are linked to an unfavorable prognosis [14].

More than 40 cancer predisposition genes are currently available for clinical genetic testing to define inherited cancer etiology and guide enhanced cancer screening for high-risk families. Majority of these are tumor suppressor genes involving various mechanisms at different levels, such as DNA repair, cell cycle regulation, apoptosis, cell proliferation, metabolism, cell adhesion, cell migration, angiogenesis, signaling passways regulation, miRNA regulator, DNA methylation, and chromatin modification. A few are proto-oncogenes whose gain-of-function mutation predisposes malignancy. Mutations in these genes may present as defined cancer or tumor predisposition syndromes or as non-syndromic hereditary cancer risk, conferring malignant risk across a broad range of organs, including but not limited to blood, brain, breast, colon, endocrine gland, eye, kidney, lung, nerve sheath, ovary, pancreas, prostate, skin, soft tissue, stomach, thyroid, urinary tract, and uterus. Most hereditary cancer risks follow an autosomal dominant inheritance pattern, with a small subset exhibiting autosomal recessive inheritance (Table 1).

## 3. Germline Genetic Testing to Identify Individuals with High Risk of Cancer

The lifetime risk of breast cancer is substantially higher in women with a hereditary predisposition. Female carriers of pathogenic variants in *BRCA1* have an estimated lifetime breast cancer risk of 60–72%, while *BRCA2* carriers face a risk of approximately 55–69% [19]. The elevated risk significantly compromises health outcomes. Early detection enables timely clinical intervention and has been shown to significantly reduce cancer-related morbidity and mortality. In the United States, breast cancer screening for women at average risk typically begins at age 40, reflecting the age when the incidence begins to rise. However, breast cancer in female BRCA1/2 carriers often occurs at a much younger age, with diagnoses reported as early as 25 years. Consequently, genetic testing to identify mutation carriers is a critical first step toward implementing appropriate surveillance and prevention strategies.

Likewise, carriers with germline CRC predisposition have a much higher risk than the general population. In the United States, the average lifetime risk of CRC is approximately 4.2–4.5%, with recommended CRC screening beginning at age 45 years [20]. In contrast, among individuals with Lynch syndrome, the cumulative incidence of CRC by age 75 is estimated to be at 46%, 43%, and 15% for mutation carriers of *MLH1*, *MSH2*, and *MSH6*, respectively. The cancer can occur as early as the third decade of life [21]. For *PMS2* carriers, the cumulative CRC risk by age 80 is estimated at 12–23% [12,22].

### 3.1. Testing Technologies in Clinical Diagnostic Laboratories

Genetic testing technologies have evolved substantially over the past decades. Before the advent of next-generation sequencing (NGS), Sanger sequencing served as the cornerstone of clinical genetic testing from approximately 2000 to 2013. DNA was extracted from lymphocytes isolated from peripheral blood, amplified by polymerase chain reaction (PCR), and analyzed by Sanger sequencing. Sanger sequencing reliably detects single-nucleotide missense and nonsense variants, as well as small insertions and deletions (a.k.a. small indels, or oligo-nucleotide deletion/insertion). However, Sanger sequencing cannot consistently identify larger deletions/duplications (dels/dups) exceeding 20–30 base pairs. To address this limitation, multiplex ligation-dependent probe amplification (MLPA), a technique specifically designed to identify large dels/dups, was introduced into clinical genetic laboratories around 2003–2004 [23]. Subsequently, between 2013 and 2014, NGS emerged as the first-tier technology for clinical cancer genetic testing, largely replacing Sanger sequencing and MLPA [24].

NGS not only enables the detection of single-nucleotide variants (SNVs) and small indels, but also larger dels/dups, including copy number variants (CNVs) at the chromosomal level. In addition, NGS technology offers superior throughput, faster turnaround times, and the ability to analyze many genes simultaneously. These advantages have reduced testing costs and greatly improved the access to clinical genetic testing for high-risk populations. Standard DNA-based NGS assays in clinical laboratories typically focus on protein-coding regions and intron-exon boundaries. As a result, variants located deep within the intron regions are not analyzed and will be missed. Deep intron variants may disrupt proper RNA splicing. Messenger RNA (mRNA) sequencing technology expanded the ability to identify these rare but clinically significant variants [25]. More recently, advances on stabilization of blood-derived RNA and messenger (mRNA) sequencing begin to be adopted by clinical laboratories. RNA sequencing also facilitates variant assessment if an exon missense variant alters normal RNA splicing.

### 3.2. Panel Genetic Testing

It has been demonstrated that multiple genes can contribute to the risk of a single cancer type, while a single gene can be associated with multiple cancer types. For example, at least nine genes are known to be associated with breast cancer risk: *ATM*, *BRCA1*, *BRCA2*, *CDH1*, *CHEK2*, *NF1*, *PTEN*, *TP53*, and *STK11* [26,27,28,29]. In the meantime, *BRCA2* is not only associated with breast cancer risk, but also the risk of the ovary, prostate and pancreas [30]. NGS enables the clinical use of multi-gene panel testing, allowing comprehensive genetic analysis without compromising turnaround time or increasing cost [31].

### 3.3. Candidate Selection to Undergo Genetic Testing for Cancer Risk

Because population-wide cancer predisposition testing is not currently feasible within existing public health resources, genetic testing is instead targeted to individuals with an elevated chance of carrying a mutation. Under current clinical practice guidelines, testing is generally recommended for individuals whose estimated probability of harboring a cancer predisposition mutation exceeds 5% [2]. Women diagnosed with breast cancer at or before age 50 or younger have an approximately 10% chance of carrying a mutation in breast cancer genes [32]. The likelihood is even higher for individuals with triple negative breast cancer (i.e., absent expression of estrogen, progesterone and HER2 receptors in cancer cells), or for males with breast cancer, regardless of age at diagnosis. These individuals are prioritized for genetic testing to establish the potential link between the cancer and a germline gene mutation [33,34]. Even though testing the affected individual provides the most informative results for the family, that person may not always be available to be tested. In such cases, NCCN guidelines recommend initiating testing in a first or second degree relative of the affected individual. Several other cancers share a genetic etiology with breast cancer. For instance, high-grade serous epithelial ovarian carcinoma, pancreatic ductal adenocarcinoma, and metastatic prostate cancer are all strongly associated with *BRCA1* or *BRCA2* mutations [30]. These cancer histories in the family all contribute to the probability of detecting *BRCA1* and *BRCA2* mutations. These histories are incorporated into the criteria for selecting individuals to undergo breast cancer genetic testing [2].

Similarly, the most common monogenic etiology for CRC predisposition is a deficiency in DNA MMR (Lynch syndrome). Early onset CRC or endometrial cancer, typically diagnosed before age 50, are primary indicators for genetic testing. In patients diagnosed at an older age, germline genetic testing is still recommended when tumor testing reveals evidence of MMR deficiency [2]. A family history of other Lynch syndrome-related cancers also increased the likelihood of detecting MMR gene mutations. MMR deficiency in tumor tissue can be assessed using two assays in clinical practice. One is immunohistochemistry (IHC) performed on formalin-fixed, paraffin-embedded tumor tissue demonstrating the absence of MMR protein expression in nuclei. The other is microsatellite stability analysis of tumor-derived DNA showing the microsatellite instability (MSI) [2].

### 3.4. Testing Samples

For germline genetic testing, sequencing DNA derived from peripheral blood lymphocytes remains the gold standard, unless an active hematologic malignancy compromises the genomic integrity when clonal hematopoiesis amplifies gene mutations arising from the bone marrow [35]. In such cases, cultured fibroblasts from biopsied normal skin tissue provide a more reliable source of unaltered germline DNA [36].

On the other hand, DNA derived from saliva or buccal swab demonstrates more than 95% concordance with blood-based sequencing results [37,38]. This high level of agreement has led to the widespread adaptation of saliva and buccal sample testing due to their convenience and comparable analytical performance relative to blood-based testing.

As a complement to DNA sequencing, RNA sequencing has increasingly been incorporated into clinical genetic testing. At present, blood collected in tubes containing RNA stabilizer remains the only standard specimen for clinical RNA-based testing.

### 3.5. Cascade Genetic Testing

Once a disease-causing pathogenic variant is identified, a first-degree relative of the carrier has a 50% probability of inheriting the same variant. Testing the first-degree relative is therefore the most logical and cost-effective approach to define the pattern of carrier status within a family. This approach is known as “cascade” genetic testing [39].

During cascade testing, it is essential to provide precise information regarding the familial genetic mutation or variant identified in the proband as documented in the clinical laboratory report. Since the early days of genetic testing, variant nomenclature has been heterogeneous, and the use of different naming systems can easily lead to confusion. Standardized variant descriptions are therefore of utmost importance for clinical care. The Human Genome Variation Society (HGVS) has promoted the use of standardized human sequence variant nomenclature since 2003. However, widespread adaptation of this standardized system in clinical laboratory reporting took more than a decade [40,41].

Genetic testing methodologies have also evolved over time. A variant detected or classified by one method may not be detected or interpreted the same way by another method. Even though concordance between NGS and Sanger sequencing exceeds 99.9% for SNV and small indels, NGS may still mis-identify rare mutations which can have important implication for clinical management [42,43]. It is therefore critically important to retain one’s genetic test report documenting the specifics of a variant identified and testing methodologies used, so that biological relatives can undergo accurate predictive genetic testing based on a reliable reference in the future.

## 4. Variant Classification

Each individual’s genome contains a substantial number of rare variants, most of which do not impair gene functions. In cancer genetic testing, numerous variants are encountered whose molecular impact or clinical significance cannot be easily determined.

Most of the familial mutations in tumor suppressor genes are unique to individual kindreds. Each tumor suppressor gene harbors a large number of distinct cancer predisposition mutations. For example, more than 1600 unique mutations have been reported in *BRCA1*, and more than 1700 in *BRCA2* [44]. Nevertheless, certain mutations occur at higher frequencies in specific racial/ethnic populations. For instance, the mutations *BRCA1* c.5266dup (a.k.a. 5382insC), *BRCA1* c.68_69del (a.k.a. 185delAG) and *BRCA2* c.5946del (a.k.a. 6174delT) have a combined prevalence of 2–3% in U.S. Ashkenazi Jews. Another mutation, *BRCA2* c.771_775del (999del5), has been found in higher rate in the Icelanders [45]. These pathogenic variants are established “founder” mutations, supported by haplotype linkage evidence indicating the origin from a common ancestor. In contrast, recurrent mutations may also be observed at increased frequencies in the general population but arise independently at vulnerable mutation hotspots without evidence of shared ancestors.

The classification of genetic variants as pathogenic or benign is nuanced, and standards can vary across clinical laboratories. Recognizing that inconsistent classification may compromise patient care, the American College of Medical Genetics and Genomics (ACMG) and the Association of Molecular Pathology (AMP) published the first guidelines of germline variant classification in 2015. The guidelines introduced a five-tier system: pathogenic, likely pathogenic, uncertain significance, likely benign, and benign [46]. The classification is based on several key criteria, including variant population frequency, DNA variant types and locations on the gene, and clinical case-level evidence [47]. Variants of lower frequency in the general healthy population but higher rate in the affected individual cohort are generally considered to have a higher probability of disease association. The potential impacts of different types of variants, such as missense, nonsense, frameshift, deletions, duplications, or splicing variants, can be predicted based on the principles of gene coding, mRNA transcription, or peptide translation. Knowledge about specific protein functional domains or tertiary structures helps to predict if the change of amino acid causes disruptions. Residues that are highly conserved across species are more likely to be functionally important, so alterations at these sites are more likely to be harmful. Computational algorithms have been developed by integrating these factors to estimate the potential impact of a variant, known as “in silico” prediction tools. Lastly, a variant segregating with disease within a kindred is more likely to be disease causing.

Despite extensive efforts, the pathogenicity of many variants remains elusive. Standardized classification criteria are often insufficient to account for the unique characteristics of specific genes or disease contexts. Clinical management demands high-quality variant interpretation and classification. To support the assessment of clinical significance of these variants, National Center for Biotechnology Information (NCBI) established ClinVar (https://www.ncbi.nlm.nih.gov/clinvar/) in 2013 as a centralized resource that archives reported variants along with their reported clinical significance [47]. In 2018, the Clinical Genome Resource (ClinGen, https://clinicalgenome.org/) [48] began to form expert curation panels for many genes and conditions. These expert work groups meet regularly to continuously review and update the variant information.

## 5. Advanced Cancer Screening

Early detection and timely treatment of cancer significantly reduce morbidity and mortality. When cancer is identified at an early or localized stage, surgical removal offers a greater chance of cure, fewer complications, and improved survival. In contrast, late-stage cancer with lymph node involvement or distant metastasis often requires systemic therapy. In these cases, recurrence rates are higher, survival is reduced, and treatment is more likely to cause toxicity to normal organs and to be poorly tolerated.

When a pathogenic variant in a cancer predisposition gene is identified, enhanced cancer screening modalities and more frequent surveillance are recommended to reduce disease-related harm. NCCN experts evaluate available data and update recommendations based on a stratified level of evidence (Table 1). Risk reduction strategies, when available, are also recommended [2]. However, despite advances in screening technologies that improve early detection, cancers may still be diagnosed at advanced stages in some individuals.

### 5.1. Breast Cancer Screening

Cancers associated with genetic risk are characterized by increased incidence and earlier age of onset. For women carrying mutations in *BRCA1* or *BRCA2*, initiating breast screening at age 25 is necessary to decrease morbidity. These recommendations are graded as NCCN Category 2A [2]. Because younger women typically have higher breast density, X-ray mammography is less sensitive to detect lesions in this population. Instead, magnetic resonance imaging (MRI) has demonstrated greater sensitivity for detecting early-stage breast cancer [49,50,51]. As a result, annual breast MRI without and with contrast is recommended beginning at age 25 as the primary advanced screening modality. Nevertheless, mammography remains superior for detecting ductal carcinoma in situ, which often manifests as pleomorphic microcalcifications without a mass or enhancement on MRI. Therefore, yearly screening mammography is added to the surveillance regimen starting at age 30 [52].

Perspective studies provided evidence that annual surveillance with MRI significantly reduced the incidence of advanced-stage breast cancer in *BRCA1* and *BRCA2* mutation carriers. However, not all cancers are detected at early stages through screening. Even within cohorts undergoing annual MRI surveillance, some advanced-stage cancers are still identified during scheduled screening, while interval cancers may arise between screening examinations. Additionally, latent or occult cancer not detected by screening may be discovered incidentally during preventive mastectomy [51,53].

### 5.2. Colorectal Cancer Screening

Genetic predisposition to CRC, such as Lynch syndrome due to mutations in *MLH1* or *MSH2*, necessitates intensive screening with full colonoscopy beginning at age 20 and repeating every one to two years (NCCN Category 2A recommendation) [2]. This screening schedule starts much earlier and is carried out more frequently than in the general population. For individuals at average risk, CRC screening typically begins at age 45, with intervals of up to 10 years depending on the findings from a previous colonoscopy [21]. Endoscopy screening and surveillance enable the detection of early-stage CRC and the identification and removal of adenomatous polyps, which are considered precancerous lesions. Enhanced endoscopic surveillance has become a cornerstone of Lynch syndrome management and has significantly improved polyp and cancer detection rates [21]. However, post-colonoscopy CRC still occurs in real-world clinical practice. These cancers are often associated with inadequate bowel preparation, incomplete examination, or the use of lower definition colonoscopes, resulting in lesions that are missed during screening but manifest clinically later.

### 5.3. Li-Fraumeni Syndrome (LFS) Screening

In some rare genetic cancer syndromes with high morbidity and mortality, such as LFS, advanced screening strategies improve outcome but are less effective compared with screening for BRCA1/2 breast cancers or Lynch syndrome CRC. LFS, caused by a germline mutation in the *TP53* gene, confers a markedly increased risk for a wide spectrum of malignancies. Tumors of the same size are more likely to exhibit aggressive behavior and rapid progression in individuals with *TP53* mutations than in those without such mutations [13,54,55]. Given the broad spectrum of malignancies and early age at onset, multi-modality cancer screening protocols have been recommended for both adults and children with LFS.

The screening protocol includes not only annual organ-specific screening, but also whole-body screening. *TP53* mutation carriers undergo annual breast MRI beginning at age 20, along with a full skin examination and complete blood count. Surveillance of the upper and lower gastrointestinal tract with endoscopy begins at age 25 and is repeated every two to five years. In addition, thorough physical examinations are emphasized. Annual whole-body MRI (WBMRI) has become a standard component of surveillance, with the goal of detecting malignant solid tumors, such as sarcomas, which may arise at any anatomical location. These are NCCN Category 2A recommendations [2].

To reduce the impact of malignancy during childhood, annual WBMRI is recommended to begin in infancy, with inclusion of the upper and lower extremities because of the elevated risk of osteosarcoma in childhood. In addition, abdominal ultrasound is recommended every 3–4 months beginning in infancy to screen for adrenocortical carcinoma.

Despite these measures, the diagnostic yield of WBMRI has been less than satisfactory. Centers with relatively large cohorts undergoing longitudinal annual surveillance have repeatedly reported missed diagnoses of cancer. WBMRI demonstrated only modest sensitivity of 43% in one study [56] and 60% in another study [54,57].

## 6. Cancer Prevention

It would be ideal if cancer could be prevented altogether; however, cancer prevention faces substantial challenges. Chemoprevention, such as using oral medications to reduce cancer risk, is an appealing strategy. But only a limited number of pharmacologic agents have been shown to effectively lower cancer incidence.

Evidence indicates that aspirin can reduce the risk of CRC in individuals with Lynch syndrome. A double-blind, randomized, international multi-center trial was conducted on a cohort of 937 individuals with Lynch syndrome. The trial demonstrated that daily aspirin at a dose of 600 mg reduced the CRC incidence from 13% to 9% [58]. This trial did not include *PMS2* mutation carriers, whose cumulative risk of CRC is lower than that of *MLH1*, *MSH2*, and *MSH6* carriers. Therefore, the benefit from aspirin is expected to be lower for *PMS2* carriers. NCCN guidelines have recommended that daily aspirin should be considered to reduce the future risk of CRC in Lynch syndrome mutation carriers, although specific benefit, risk, adverse effects, and childbearing plans should be carefully evaluated for each individual (NCCN recommendation based on high consensus). Further evidence is also needed to determine whether aspirin therapy can reduce the need for early and frequent colonoscopy currently recommended for these patients.

Epidemiological studies demonstrated that oral contraceptives can significantly reduce ovarian cancer among average women in the general population with a long-term effect [59]. In BRCA1/2 mutation carriers, oral contraceptive use also demonstrated a nearly 50% reduction in ovarian cancer. A recent international retrospective study demonstrated a hazard ratio of 0.37 when the duration of use is 10 years or longer, and the protective effects persisted for more than 15 years [60]. However, the clinical utility of this preventive approach depends on whether the residual cancer risk can be further mitigated through additional strategies.

Overall, chemoprevention for cancer with genetic predisposition remains under-investigated. A more definitive, though drastic, preventive approach is risk-reducing surgery, which involves removing at-risk organs before cancer develops. Preventive surgery has proven to be highly effective in lowering cancer-related mortality. Preventive mastectomy is an established and accepted option for women carrying *BRCA1* and *BRCA2* mutations [2,61]. For women at high risk of ovarian cancer, such as BRCA1/2 carriers, available screening methods have not been shown to effectively reduce mortality. Even with comprehensive screening, up to 80% of the cancers were diagnosed in advanced stages [62]. As a result, risk-reducing surgery has become the standard preventive strategy. A significant portion (48–71%) of high-grade serious ovarian cancer originates in the fallopian tubes [63], making risk-reducing surgery inclusive of both fallopian tubes and ovaries, i.e., salpingo-oophorectomy. The risk of ovarian cancer begins to rise in the late 30s to early 40s for *BRCA1* carriers, and approximately 10 years later for *BRCA2* carriers [61]. The ovaries play a vital role in premenopausal women, not only in fertility and family planning, but also in maintaining overall health through the production of female hormones. Since risk-reducing surgery is currently the only effective strategy to prevent ovarian cancer, its timing must carefully balance the benefits of cancer prevention with the consequences of ovarian hormone loss.

In some hereditary cancer predisposition conditions, enhanced screening may reveal an alarmingly high burden of precancerous lesions, making individual lesion removal impractical. In individuals with Familial Adenomatous Polyposis (FAP, classical type), polyps can appear as early as age 8. The proliferating adenomatous polyps can number in the hundreds or even thousands, carpeting the colon mucosa, destined to progress into cancer. In these cases, the only way to prevent colorectal cancer and preserve life is through complete removal of the colon.

## 7. Hereditary Tumor Syndromes

Dysfunction of a single gene can lead to a wide range of clinical manifestations. A genetic syndrome refers to a constellation of features affecting multiple tissues, organs, or physiological functions that arise from a shared underlying genetic cause. Some hereditary tumor syndromes not only confer an increased risk of malignancy but also predispose individuals to tumors that are histologically benign yet clinically aggressive. In addition to cancer prevention, screening and treatment, additional syndrome-related dysfunctions also require medical attention.

PTEN hamartomatous tumor syndrome (PHTS) is known for an increased cancer risk of the breast, endometrium of the uterus, thyroid, colon, and kidneys. Moreover, hamartomatous growths, the hallmark of PHTS, are histologically benign lesions characterized by disorganized overgrowth of mature tissues which may begin to develop in childhood. These hamartomatous lesions can manifest as gastrointestinal polyposis, large lipomas, dysplastic cerebellar gangliocytoma (Lhermitte–Duclos disease), or vascular malformations. Benign thyroid growth, such as a goiter, is also a part of the disease spectrum. In many cases, the size or number of these hamartomas can be substantial enough to require clinical intervention to alleviate symptoms [64]. Other manifestations, including larger head size (macrocephaly), autism spectrum disorder, and developmental delay, can present in early childhood and require evaluation, ongoing surveillance, and sometimes intervention.

Several other hereditary cancer and tumor syndromes are also well characterized and established clinical management recommendations are available. Examples include neurofibromatosis type 1 (NF1), tuberous sclerosis complex (TSC), von Hippel–Lindau disease (VHL), and *DICER1* tumor predisposition syndrome.

Genes responsible for hereditary tumor syndromes are commonly included in cancer genetic testing panels. When a pathogenic variant is identified and a tumor syndrome diagnosis is established, comprehensive screening, surveillance, and intervention strategies must be implemented to address the full spectrum of manifestations.

Autosomal dominant inheritance means a monoallelic mutation in a single copy of a gene, passed down from either the maternal or paternal side, is sufficient to confer increased cancer risk. However, when both parents are carriers of a mutation on the same gene, each child has a one-in-four chance of inheriting pathogenic variants on both copies of the gene (biallelic mutations), which in turn result in severe childhood-onset conditions. One such example is Constitutional Mismatch Repair Deficiency (CMMRD), a.k.a. Biallelic Mismatch Repair Deficiency (BMMRD), a rare autosomal recessive cancer syndrome. When a child inherits mutations in both copies of a Lynch syndrome gene, profound MMR deficiency develops, often becoming evident in infancy or early childhood. The affected child has a markedly increased risk of developing colorectal polyps, colorectal cancer, brain tumors, and leukemia, which significantly limit life expectancy [65]. When counseling families with MMR gene mutations, this rare possibility of CMMRD should be discussed, along with the availability for reproductive planning. CMMRD is most observed in families carrying *PMS2* mutations, as *PMS2* carriers are more prevalent in general population than other MMR genes. Additionally, *PMS2* family histories are often not strongly suggestive of Lynch syndrome because cancer penetrance related to this gene is relatively modest.

There are additional autosomal recessive childhood conditions related to cancer predisposition genes. Examples include ataxia-telangiectasia caused by biallelic *ATM* mutations and Fanconi anemia resulting from biallelic mutations in homologous recombinant repair genes, such as *BRCA2*, *BRIP1*, *PALB2*, and *RAD51C*.

## 8. Incomplete Penetrance

Genetic penetrance refers to the probability that an individual carrying a pathogenic variant will develop the associated disease phenotype. Complete penetrance indicates a 100% likelihood that the condition will manifest over a lifetime. A monogenic cancer predisposition syndrome is defined by the presence of a single germline mutation that confers a significantly increased risk of cancer. The magnitude of this risk varies depending on the cancer type, the specific gene involved, and even a specific variant within the same gene.

A truncating mutation is a genetic change that introduces a downstream premature stop codon, triggering mRNA degradation, a process called nonsense-mediated mRNA decay (NMD). Among carriers with a *BRCA1* truncating mutation, the lifetime risk of breast cancer is markedly increased to approximately 66–75%, representing about a 12.8-fold increase over the general population and reflecting a high penetrance. In comparison, *BRCA1* missense variants are generally associated with a lower risk, an estimated 3.9-fold over the population average [33]. Furthermore, certain variants confer particularly reduced risks, such as *BRCA1* c.5096G>A (p.Arg1699Gln); its associated risk is a 2.8-fold increase over the average [66]. Such variants are often described as “hypomorphic”, “reduced penetrance”, or “intermediate risk”, as their associated risks are substantially lower than those conferred by other pathogenic variants in the same gene.

The cancer risk can vary significantly depending on the genes involved. The lifetime risk of breast cancer associated with some genes is higher than that of the general population, but lower than that observed in BRCA1/2 carriers. Applying uniformed screening and prevention guidelines designed for high-risk genes to those with moderate-risk genes may result in harm due to over-screening or over-treatment. Mutations in the *CHEK2* gene, for example, confer a moderate increase in breast cancer risk, with an estimated lifetime risk of 23–27% [67]. For *CHEK2* carriers, sufficient evidence supports tailoring screening recommendations to reflect the moderate risk and relatively later age of onset. Current guidelines recommend that annual breast screening begin at age 30–35 years, and the associated risk is generally not high enough to warrant a preventive risk-reducing mastectomy [2].

For Lynch syndrome, large cohort studies have provided refined genotype-specific insights into cancer characteristics, including cancer incidence rates and age at onset. Long-term follow-up of Lynch syndrome carriers has demonstrated that mutations in *MSH6* and *PMS2* are associated with a relatively lower cancer risk compared with mutations in *MLH1* and *MSH2* [68]. Accordingly, the recommended age to initiate colonoscopy screening for individuals with *MSH6* or *PMS2* mutations has been deferred to 30–35 years, instead of 20–25 years for *MLH1* and *MSH2* [2].

## 9. Polygenic Risks of Cancer

The cause of cancer is multifactorial. The penetrance of a genetic risk phenotype is influenced by the specific gene, type and location of the mutation, family history, age, genetic modifiers such as polygenic risk scores (PRS), epigenetics, and other environmental exposures.

Single-nucleotide polymorphisms (SNPs) are single-nucleotide substitutions in the DNA sequence at a specific genomic location. SNPs account for 94% of DNA replication errors, making them the dominant form of human genetic diversity, responsible for individual differences in physical traits and sometimes disease susceptibility. Genome-wide association studies (GWAS) leverage high-throughput genotyping technologies to test hundreds of thousands to millions of SNPs across the genomes of large populations, identifying associations between genetic variants and disease phenotypes [69]. Approximately 5% of disease-associating SNPs, also called risk alleles, are located in the coding regions which may directly affect gene function. The remaining majority (95%) of the SNPs are found in non-coding regions, where they may influence disease risk by modulating various regulatory steps that affect gene expression [70]. GWAS characterizes the weight (i.e., effect size) of each risk allele. Polygenic risk scores (PRS) for a disease can be calculated using a weighted sum of risk alleles.

In the absence of high or moderate risk monogenic cancer risk mutations, many common genetic variants (SNPs or risk alleles) each contribute a small influence on cancer risk. The cumulative effect of these variants can determine an individual’s overall susceptibility. Unlike rare and high penetrance mutations in genes such as *BRCA1* or *BRCA2* which dramatically elevate cancer risk, common variants typically have modest effects (odds ratios of 1.1–1.5 per allele). Since the first cancer GWAS in 2007, thousands of such common risk alleles have been identified across multiple cancer types. Consequently, polygenic risk scores (PRS) have been developed and studied for numerous cancer types to quantify risk [71,72]. To date, PRS for breast, prostate, and colorectal cancers has demonstrated the strongest clinical validity and predictive performance.

The influence of common genetic variants, or SNPs, is also evident when they coexist with moderate risk gene mutations. For example, in *CHEK2* mutation carriers, common SNPs can further modify breast cancer risk, expanding the estimated lifetime risk range from 9% to 49% [73].

## 10. Somatic Tumor Genetic Testing

Beyond germline genetic testing, advances in next-generation sequencing (NGS) have uncovered the complex and dynamic clonal genomic alterations in malignant tumors. Unlike germline mutations, which originate at conception and are faithfully replicated in every cell division, somatic changes arise in subpopulations of cells due to defects in genome maintenance or failures in DNA repair mechanisms. Before the DNA molecular composition of cancers was understood, treatment decisions were based solely on tumor histology. With NGS molecular profiling, some cancer therapy can now be tailored for individual patients according to actionable genetic alterations in tumor cells. Such customized therapy can help to avoid ineffective treatment, maximize therapeutic efficacy, and reduce toxicity [74].

Genomic profiling of tumors not only identifies somatic DNA alterations but may also reveal germline pathogenic variants if they are preserved in tumor cells or in adjacent normal cells. The incidental revelation of hereditary cancer risk is important not only for the individual undergoing treatment, highlighting potential risks for other cancers, but also for their biological relatives, who may face an elevated cancer risk.

It is important to note that NGS of tumor tissue, while designed to detect actionable somatic mutations, may not identify all germline mutations. Studies suggest that approximately 10% of germline mutations can be missed by tumor NGS testing [75]. Paired genetic testing, using both tumor tissue and normal tissue, such as peripheral blood lymphocytes, provides a more comprehensive view of genomic alterations [76].

## 11. Targeted Therapy and Germline Cancer Risk Genes

Because of the unique molecular mechanisms, therapies routinely used for sporadic cancers may not be suitable for certain hereditary cancers. For instance, individuals with Li-Fraumeni syndrome (LFS) who carry *TP53* germline mutations are more vulnerable to developing secondary malignancies following radiation exposure to normal tissues during prior cancer treatment [77,78,79]. Consequently, current clinical guidelines recommend avoiding therapeutic radiation whenever possible [2].

On the positive side, understanding of tumor suppressor gene function has enabled the exploitation of tumor-specific molecular mechanisms, leading to the development of targeted cancer therapies for individuals with hereditary cancer predisposition.

The most extensively studied targeted therapies are those for BRCA1/2-associated cancers. Poly (ADP-ribose) polymerase (PARP) provides a mechanism in single-strand DNA repair. Cancer cells that are already deficient in homologous recombination DNA repair, such as those with *BRCA1* or *BRCA2* mutations, are dependent on PARP-mediated alternative repair pathways for survival. Inhibition of PARP therefore creates synthetic lethality for these tumors [80]. The U.S. Food and Drug Administration (FDA) has approved several PARP inhibitors for the treatment of breast and ovarian cancer with germline *BRCA1* or *BRCA2* mutations.

Mismatch repair deficiency results in a high neoantigen burden within tumor cells, making these tumors sensitive to immune checkpoint blockade therapy. By blocking programmed death-1 (PD-1) receptor on T cells, PD-1 inhibitors showed significant clinical efficacy in cancer with MMR deficiency [81]. Two PD-1 inhibitors have been approved by the FDA for microsatellite instability-high or MMR-deficient (MSI-H, dMMR) solid tumors that have progressed following prior treatment. Notably, this indication extends beyond Lynch syndrome cancers to include tumors with MMR deficiency arising from somatic alterations.

## 12. Genetic Information Nondiscrimination Act (GINA)

Germline cancer predisposition in otherwise healthy individuals may raise concerns about genetic discrimination. In the United States, the Genetic Information Nondiscrimination Act (GINA) was enacted in 2008. GINA prohibits health insurers and employers from using genetic information, including genetic test results and family history, in making decisions about coverage, premiums, hiring, firing, or promotion. However, GINA has limitations. The law does not extend protection against discrimination in life insurance, disability insurance, or long-term care insurance. Additionally, it also does not apply to employers with fewer than 15 employees, members of the military (who have separate protections through Tricare and Veterans Affairs), federal employees, or the Indian Health Service. Importantly, GINA protects only asymptomatic individuals who test positive for cancer predisposition or have a family history of genetic predisposition (GIM statement) [82].

## 13. Discussion

Genetic testing for germline cancer risk has made tremendous strides in facilitating precision cancer prevention, screening and treatment. Nevertheless, continued efforts are needed in several key areas.

Variant classification remains a major challenge. Owing to the heterogeneity of the human genome and the abundance of rare, private variants, multigene cancer panel testing frequently identifies VUS. A substantial number of individuals undergoing germline multi-gene panel testing were found to carry at least one VUS, with reported rates ranging from 8% to 28%. These rates vary depending on interpretation criteria, the patient cohort, and the number of genes analyzed [83,84,85]. Although 89% to 91% of VUS are eventually reclassified as benign or likely benign, their initial identification can create significant clinical management dilemmas [83,86,87]. To enhance confidence in variant classification and reduce the proportion of VUS, efforts have been focused on several areas. One approach is the integration of germline RNA sequencing into variant classification [25,87]. Others involve gene-targeted strategies, including in vitro functional analyses, for example, Homology-Directed Repair (HDR) assay to resolve *BRCA2* VUS [88]. For Lynch syndrome, the well-calibrated and validated cell-free in vitro MMR activity (CIMRA) assay has been the functional assay recognized by ACMG/AMP for all four MMR genes [89]. The growing volume of tumor somatic variant data provides additional opportunities to clarify variant impact. To evaluate such potential, ClinGen Germline/Somatic Variant Subcommittee (GSVS) investigated somatic data resources, repositories and studies, then conducted integrative curation exercises. With that, GSVS was able to assign evidence strength to several criteria. Tumor RNA sequencing data can provide strong evidence for splicing pathogenicity, tumor mutational signatures provide supporting evidence for pathogenicity, while somatic hotspots provide moderate evidence for pathogenicity [90]. Gene-specific variant curation expert panels continuously seek to improve the accuracy and resolve uncertainty. For example, the BRCA1/2 classification subgroup applied statistical methods to calibrate the strength of the broad classification criteria defined by ACMG/AMP guidelines [91].

Variable penetrance of cancer risk associated with specific genes can create clinical dilemmas. A one-size-fits-all approach can lead to over-screening and excessive intervention or, conversely, under-screening and insufficient intervention. Beyond the recognized modifying power of PRS in carriers of moderate-risk gene mutations, such as *CHEK2*, cancer risks conferred by high-risk genes have also been shown to be influenced by SNP-based PRS. While some SNPs impact cancer risk both in the general population and in high-risk individuals, other SNP loci only modify risk in specific gene mutation carriers, such as BRCA1/2 [92]. In addition, the strength of PRS modification varies across age groups, highlighting its potential not only to refine individualized risk estimates but also to improve the prediction of age at cancer onset [93].

Cancer surveillance in Li-Fraumeni syndrome presents significant challenges. Individuals with LFS face a lifelong cancer risk beginning in childhood, necessitating broad and intensive surveillance strategies. This burden of surveillance often results in “screening fatigue”. Moreover, despite extensive effort, a substantial proportion of cancers are still detected at advanced stages. Emerging research suggests that liquid biopsy may enhance early cancer detection in LFS. A proof-of-principle study has demonstrated the feasibility of a multimodal liquid biopsy approach for early detection. This method combining a targeted gene panel, shallow whole-genome, and cell-free methylated DNA immunoprecipitation sequencing, was tested in a longitudinal cohort of 89 LFS patients. The results showed earlier detection when compared with the current clinical surveillance methods, demonstrating its promising role of increasing the accessibility and sensitivity in LFS cancer detection. Currently, liquid biopsy is considered an adjunct to existing surveillance protocols rather than a replacement [94].

## Figures and Tables

**Table 1:** High-penetrance cancer syndromes: related genes, mechanisms, typical cancers, first-line surveillance, risk-reduction options.

Cancer Syndromes ^a^	Causative Genes	Typical Cancers and Features ^b^	Primary Screening and Surveillance ^c,d^	Risk Reduction Options ^c^	Major Mechanisms of Encoded Protein ^e^
Hereditary Breast and Ovarian Cancer	*BRCA1*, *BRCA2*, *PALB2*	Breast (including male breast), ovary (including fallopian tube and primary peritoneal) ^f^, pancreas ^g^ (for BRCA1/2 only), and prostate (for BRCA1/2 only) cancers.	Early and advanced breast imaging screening. Early prostate screening (for BRCA1/2 only) with PSA. Pancreas imaging screening if there is a family history of pancreatic cancer (for BRCA1/2 only).	Mastectomy, Salpingo-oophorectomy (SO) at age 35–50, depending on the gene and family planning.	Core components controlling cell cycle checkpoints (CCC) and conducting homologous recombinant repair (HRR) triggered by DNA double-strand break (DSB).
Hereditary Ovarian Cancer	*BRIP1*, *RAD51C*, *RAD51D*	Ovarian (including fallopian tube and primary peritoneal) cancer. Breast cancer for RAD51C/D.	Advanced breast imaging screening for RAD51C/D only.	SO at age 45–50.	RAD51C/D: Regulators in protein complexes for DNA DSB HHR and CCC control; *BRIP1*: A DNA helicase in DNA DSB HRR, CCC control, and interacts with BRCA1.
Hereditary Breast Cancer	*ATM*, *BARD1*, *CHEK2*	Breast cancer. Pancreatic cancer for *ATM* only.	Early and advanced breast imaging screening. Pancreas imaging screening (for *ATM* only) if there is a family history of pancreatic cancer.		ATM: A kinase in multiple CCCs and responses to DNA DSB repair; BARD1 binds to BRCA1 protein, participates in HRR and regulates apoptosis; CHEK2: A kinase in multiple CCCs and regulating responses to DNA damage and apoptosis.
Li-Fraumeni Syndrome (LFS)	*TP53*	Breast, soft tissue sarcoma, osteosarcoma, gastrointestinal, brain, and adrenocortical cancers, melanoma and leukemia.	Early and advanced screening including total body MRI, breast imaging, upper and lower GI tract endoscopy.	Preventive mastectomy.	A master tumor suppressor regulating cellular responses to stress, DNA damage, oncogene activation, cell cycle arrest, apoptosis, senescence, and metabolic adaptation.
PTEN Hamartoma Tumor Syndrome (PHTS)/Cowden Syndrome	*PTEN*	Breast, thyroid, colorectal (CRC), endometrial and renal cell (RCC) cancers.	Early and advanced imaging screening for breast, thyroid, and kidney. Early and frequent endoscopy screening for CRC. Endometrial clinical surveillance and/or biopsy.	Preventive mastectomy.	Negative regulator for the PI3K/AKT/mTOR signaling pathway to regulate cellular metabolism, cell proliferation, and survival.
Peutz-Jeghers Syndrome	*STK11*	Breast, pancreatic, CRC, upper GI tract, endometrial, cervical (minimal deviation adenocarcinoma) cancers. Benign ovary-sex cord tumor with annular tubules (SCTAT) causing precocious puberty, testes Sertoli cell tumors causing feminization. Perioral freckles.	Early and advanced imaging screening for breast, pancreas, cervix. Endoscopy screening for upper and lower GI tract.	Preventive mastectomy. Preventive hysterectomy with individualized timing.	Sensing energy stress to switch on catabolic pathways to generate ATP and activate TSC2.
Hereditary Diffuse Gastric Cancer	*CDH1*	Breast and gastric cancers.	Early and advanced breast imaging screening. Upper endoscopic screening with random biopsies.	Preventive mastectomy. Preventive gastrectomy.	E-cadherin is a cell–cell adhesion molecule. It also regulates signal transduction, cell migration and invasion.
Lynch Syndrome	*MLH1*, *MSH2*, *MSH6*, *PMS2* ^h^	CRC and endometrial cancers. Other cancer risks (except for *PMS2*): ovarian, pancreatic, gastric, duodenal, sebaceous adenocarcinomas, and upper tract urothelial cancers.	Early and frequent endoscopic screening for colon. Screening for upper GI tract (except for *PMS2*). Endometrial surveillance and/or biopsy. Full Skin examination (except for *PMS2*).	Preventive hysterectomy and SO are considered with individualized timing. Aspirin CRC chemoprevention balancing individualized benefit and risk.	DNA mismatch repair during DNA replication.
Adenomatous Polyposis	*APC*	Adenomatosis polyposis, CRC, duodenal, gastric, and thyroid cancers.	Early and frequent endoscopic screening of upper and lower GI tract, thyroid ultrasound screening beginning in late teens. Colonoscopy beginning at age 10–15.	Preventive colectomy when polyp burden is high.	Regulates the Wnt/β-catenin signaling pathway to control cell proliferation, survival, and oncogene expression. It also controls cell migration, adhesion, and chromosome stability.
Juvenile Polyposis Syndrome	*BMPR1A*, *SMAD4*	Juvenile polyposis ^i^ and CRC. Stomach cancer for *SMAD4* only.	Early (beginning at age 12–15) and frequent endoscopic screening for upper (*SMAD4* only) and lower GI tract.	Preventive colectomy if polyp burden is high.	BMPR1A: A kinase receptor that transduces signals to active the SMAD1/5/8 signaling pathway to regulate cell proliferation, differentiation and apoptosis. SMAD4 mediates TGF-β and BMP signaling pathways, induces apoptosis, inhibits cell growth and angiogenesis.
Autosomal Recessive Polyposis	*MUTYH*, *NTHL1*	Biallelic mutations predispose adenomatous polyposis and CRC. Biallelic *MUTYH* mutations also increase duodenal cancer risk.	Early and frequent endoscopic screening of upper (*MUTYH* only) and lower GI tract.	Preventive colectomy if polyp burden is high.	MUTYH: A part of the base excision repair (BER) mechanism in response to oxidative DNA damage. Deficient MUTYH leads to APC inactivation and KRAS proto-oncogene activation. NTHL1: A part of the BER in response to oxidative DNA damage.
Other Adenomatous Polyposis Syndrome	*POLD1*, *POLE*	Adenomatous polyposis. CRC and duodenal cancers.	Early and frequent endoscopic screening of upper and lower GI tract.	Preventive colectomy if polyp burden is high.	Subunits of the DNA polymerase responsible for excising mis-paired bases during DNA replication.
Birt-Hogg-Dubé syndrome	*FLCN*	Renal cell cancer (RCC) of chromophobe, hybrid oncocytoma, clear cell, or papillary type. Skin papules (fibrofolliculomas), lung cysts and spontaneous pneumothorax.	Advanced kidney imaging screening.		A GTPase-activating protein (GAP) that senses nutrients and modulates mTORC1 and AMPK signaling.
Hereditary Leiomyomatosis and Renal Cell Cancer (HLRCC)	*FH*	FH-deficient RCC. Leiomyomatosis of skin and uterus.	Advanced kidney imaging screening, beginning at age 8–10.		A TCA cycle enzyme. Absence of FH leads to fumarate (oncometabolite) accumulation which activates multiple oncogenic cascades, including metabolic reprogramming to aerobic glycolysis (the Warburg effect).
Von Hippel–Lindau Syndrome	*VHL*	Multifocal clear cell RCC, pheochromocytoma (PCC), hemangioblastoma in brain/spine and retina, pancreatic neuroendocrine tumor (panNET). Pancreatic cysts, endolymphatic sac tumors.	Advanced kidney and abdomen imaging screening beginning at age 15. Brian and spinal cord imaging screening at age 11. PCC screening with metanephrines at age 5. Multisystemic screening and surveillance targeting organs at risk.		A part of the VCB-CUL2 E3 protein complex in oxygen-sensing pathway. The loss of VHL activates HIF-dependent tumorigenesis via angiogenesis, proliferation, and metabolic reprogramming.
Hereditary Paraganglioma and Pheochromocytoma	*SDHA*, *SDHAF2*, *SDHB*, *SDHC*, *SDHD*	Paraganglioma and PCC with metastatic potential, SDH-deficient RCC, and gastrointestinal stromal tumor (GIST)	Early and advanced imaging from skull base to pelvis (such as MRI) and biochemical markers (plasma free metanephrines), beginning as early as age 6–10, depending on the penetrance of the gene. Screening and interval are modified for less penetrance genes, such as *SDHA*.		Subunits of the succinate dehydrogenase (SDH) (mitochondrial complex II) catalyzing the oxidation of succinate to fumarate. Absence of SDH complex leads to succinate (oncometabolite) accumulation and drives tumorigenesis via multiple interconnected mechanisms, including global hypermethylation and metabolic reprogramming.
Hereditary Paraganglioma and Pheochromocytoma	*TMEM127*	Paraganglioma and PCC with metastatic potential, RCC	Early and advanced imaging from skull base to pelvis (such as MRI), metanephrines, beginning at age 10–15.		A negative regulator of mTORC1 signaling. It also regulates tyrosine kinase (such as RET) trafficking and degradation.
Hereditary Pheochromocytoma	*MAX*	PCC with metastatic potential	Early and advanced imaging of abdomen (such as MRI) and metanephrines, beginning at age 8–10.		A part of the MYC-MAX-MXD transcriptional network. It represses *MYC* oncogene driven transcription activation.
Multiple Endocrine Neoplasia Type 2 (MEN2A, MEN2B)	*RET*	Medullary thyroid carcinoma, PCC, parathyroid adenoma/hyperplasia. Features of MEN2B also include intestinal ganglioneuromas, mucosal neuromas, and Marfanoid habitus.	Thyroid and neck imaging screening. Surveillance with biochemical markers relevant to endocrine glands at risk. Surveillance begins at early childhood till preteen years, depending on the mutation penetrance and glands at risk.	Preventive total thyroidectomy for high-risk *RET* mutations at early childhood.	A proto-oncogene encoding a receptor tyrosine kinase. The activating point mutations result in constitutive RET activation and several downstream signaling pathways promoting cell proliferation, survival and migration.
Multiple Endocrine Neoplasia Type 1 (MEN1)	*MEN1*	Duodenal/pancreatic/gastric neuroendocrine tumors (NETs), parathyroid adenoma/hyperplasia, pituitary adenomas, lung/thymic carcinoids, angiofibromas, collangiomas, lipomas, and meningiomas.	Surveillance of biochemical markers relevant to endocrine glands at risk. Surveillance imaging includes pituitary, chest, abdomen, and pelvis. Surveillance beginning at or before age 15.		Menin is a scaffold protein interacting with chromatin-modifying complexes and suppressing several signaling pathways to regulate gene expression, genome stability and cell proliferation.
Neurofibromatosis type 1 (NF1) ^j^	*NF1*	Malignant peripheral nerve sheath tumor (MPNST), GIST, breast cancer, optic glioma, brain tumors, PCC, cutaneous and internal plexiform neurofibromas, café-au lait macules and axillary freckling, Lisch nodules, vasculopathy.	Early and advanced breast imaging screening. Vision and ophthalmology surveillance beginning in infancy. Multisystemic screening and surveillance targeting organs at risk.		Neurofibromin facilitates RAS GTPase-activating protein (GAP) to down-regulate RAS-MAPK signaling cascade.
Tuberous Sclerosis Complex	*TSC1*, *TSC2*	Clear cell RCC, renal angiomyolipoma, cardiac rhabdomyoma, subependymal giant cell astrocytoma (SEGA), brain cortical dysplasia/tubers, seizures, facial angiofibromas, ungual fibromas, lymphangioleiomyomatosis (LAM) in the lungs, Shagreen patch, hypomelanotic macules.	Advanced kidney imaging screening beginning at age 12, brain MRI, EEG and cardiac screening in infancy, lung imaging screening for females. Multisystemic evaluation screening and surveillance targeting organs at risk.		Integrating signals from multiple pathways to modulate mTORC1 activity to inhibit anabolic synthesis of protein, lipid, and nucleotide, as well as inhibit catabolism.
Hereditary Melanoma Syndromes	*CDKN2A*, *BAP1*	*CDKN2A*: pancreatic cancer and melanoma. *BAP1*: clear cell RCC, malignant uveal melanoma, cutaneous melanoma, and mesothelioma.	Regular and frequent full skin examination. Pancreatic imaging screening. Ocular examination, chest and abdomen imaging (for *BAP1* only).		*CDKN2A* encodes two independent tumor suppressors, p16INK4a and p14ARF, responsible for cell cycle arrest and apoptosis. *BAP1* participates in epigenomic integrity maintenance by chromatin regulation, DNA repair, and apoptotic signaling.
Hereditary Melanoma	*CDK4*	Atypical multiple moles and cutaneous melanoma.	Regular full skin examination.		A cyclin-dependent kinase and proto-oncogene. Rare activating missense mutations on *CDK4* result in constitutive activation to drive cell cycle progression.
Nevoid Basal Cell Carcinoma Syndrome/Gorlin syndrome	*PTCH1*	Primary multifocal basal cell carcinoma on skin, jaw keratocyst.	Regular full skin examination beginning in childhood. Dental surveillance beginning in infancy.		A receptor participating in suppression of the G-protein-coupled receptor Smoothened (SMO) and preventing downstream signaling.
DICER1 Tumor Predisposition Syndrome	*DICER1*	Differentiated thyroid cancer, pleuropulmonary blastoma, Sertoli-Leydig cell tumor, including gynandroblastoma, and pediatric cystic nephroma. Lung cysts and thyroid nodules.	Pulmonary clinical and imaging screen beginning at birth. Thyroid imaging screen beginning at age 8 years. Other surveillance targeting organs at risk.		An RNase III endoribonuclease essential for miRNA maturation. Absence of DICER1 leads to loss of tumor-suppressive 5p-miRNAs and gain of oncogenic 3p-miRNAs.

^a^ All hereditary risks follow autosomal dominant inheritance unless it is otherwise specified. ^b^ The evidence of gene and cancer association is categorized as “Strong” or “Very Strong” based on NCCN guidelines accessed 24 April 2026. ^c^ Screening, surveillance, and risk reduction: the strength of evidence for all recommendations is “Category 2A” in NCCN guidelines accessed 24 April 2026. When not available in NCCN, the recommendations are based on GeneReviews^®^, the level of evidence of which is generally regarded as “Expert opinion”. (In [15]). ^d^ The beginning age for screening and surveillance is specified if it is recommended before adulthood. ^e^ All genes are tumor suppressors unless it is specified as “proto-oncogene”. ^f^ Ovarian cancer is of the epithelial type unless it is otherwise specified. ^g^ Pancreatic cancer is ductal adenocarcinoma unless it is otherwise specified. ^h^ For Lynch syndrome genes, *EPCAM* is not included because it is not a mismatch repair gene, but its 3′ deletion prohibits a normal *MSH2* gene from being transcribed. ^i^ Juvenile polyposis: “juvenile” refers to the histological features instead of age at onset. ^j^ NF1: MRI surveillance recommendation is categorized as “Weak evidence”—expert majority decision WITHOUT consistent evidence. Breast cancer screening has “Moderate evidence”—moderate expert consensus WITH inconsistent evidence AND/OR new evidence likely to support the recommendation (custom grading scale developed specifically by ERN GENTURIS, necessity for rare disease such as NF1). References: [16,17,18].

## Data Availability

No new data were created or analyzed in this study. Data sharing is not applicable to this article.
